# Ultraviolet B-induced expression of amphiregulin and growth differentiation factor 15 in human lens epithelial cells

**Published:** 2011-01-13

**Authors:** Hiromi Osada, Yoshino Yoshitake, Takayuki Ikeda, Yasuhito Ishigaki, Takanobu Takata, Naohisa Tomosugi, Hiroshi Sasaki, Hideto Yonekura

**Affiliations:** 1 Department of Ophthalmology, Kanazawa Medical University School of Medicine, Uchinada, Ishikawa, Japan; 2 Department of Biochemistry, Kanazawa Medical University School of Medicine, Uchinada, Ishikawa, Japan; 3 Medical Research Institute, Kanazawa Medical University, Uchinada, Ishikawa, Japan

## Abstract

**Purpose:**

Epidemiological and experimental studies have revealed that exposure to ultraviolet B (UVB) light can induce cataractogenesis. The objective of this study was to determine gene expression changes in human lens epithelial cells in response to UVB exposure and identify factors that can be involved in UVB-induced cataractogenesis.

**Methods:**

SV40 T-antigen-transformed human lens epithelial cells (SRA01/04) were irradiated at various UVB-energy levels (10–80 mJ/cm^2^) and checked for viability. An irradiation condition of 30 mJ/cm^2^ was adopted for transcriptome analysis. Total RNAs isolated from UVB-exposed and unexposed cells at 12 h and 24 h after UVB exposure were examined for global gene expression changes using Affymetrix Human Gene 1.0 ST array. mRNA levels of specific genes were examined by RT–PCR and real-time PCR, and protein levels in the conditioned media were assayed by ELISA. To examine mRNA expression in human lens, primary cultured human lens epithelial (HLE) cells were prepared from surgically removed lens epithelium, and used for UVB-irradiation and expression analysis. Effects of certain gene products on SRA01/04 cell metabolism were examined using commercially available recombinant proteins.

**Results:**

Expression of most the genes analyzed was essentially unchanged (between 0.5 and 2.0 fold) in UVB-irradiated cells compared to non-irradiated cells at both 12 and 24 h after UVB exposure. Sixty one and 44 genes were upregulated more than twofold by UVB exposure at 12 h and 24 h, respectively. Emphasis was placed on genes encoding extracellular proteins, especially growth factors and cytokines. A total of 18 secreted protein genes were upregulated more than twofold at either or both time points. Amphiregulin (*AREG*) and growth differentiation factor 15 (*GDF15*) were chosen because of their higher upregulation and novelty, and their upregulation was confirmed in SRA01/04 cells using RT–PCR and real-time PCR analysis. AREG and GDF15 protein levels in conditioned media significantly increased at all UVB-energy points at 24 h, while they were scarcely detectable at 12 h. *AREG* and *GDF15* mRNA levels were also significantly upregulated in UVB-irradiated primary cultured HLE cells compared with the corresponding control culture. AREG significantly stimulated ^3^H-thymidine and ^3^H-leucine uptake in SRA01/04 cells as did a positive control epidermal growth factor (EGF). Recombinant GDF15 did not stimulate ^3^H-thymidine incorporation at any concentration tested, but significantly stimulated ^3^H-leucine uptake. RT–PCR analysis demonstrated that primary cultured HLE and SRA01/04 cells expressed not only epidermal growth factor receptor (*EGFR*) mRNA but also transforming growth factor β receptors (*TGFBR1* and *TGFBR2*) mRNAs.

**Conclusions:**

These results indicate that AREG and GDF15 produced in response to UVB exposure can affect the growth and protein synthesis of lens epithelial cells, suggesting that they have autocrine and paracrine roles related to pathological changes of lens tissue during long-term UVB exposure.

## Introduction

Solar ultraviolet (UV) radiation contains wavelengths from approximately 200 to 400 nm but only ultraviolet B (UVB; 290–320 nm) and ultraviolet A (UVA; 320–400 nm) reach the terrestrial surface. Depletion of ozone increases the levels of UV radiation, particularly UVB, reaching the Earth’s surface [1]. Exposure to solar UV radiation has been implicated in a spectrum of skin and ocular pathologies.

Cataracts are the main cause of human blindness worldwide, responsible for 48% of the total cases of blindness [2]. Increasing evidence suggests that exposure of the eye to UVB irradiation may cause cortical and posterior subcapsular cataract in humans and animals [3-6]. Characteristic features include abnormal cortical fiber migration, swelling, and intracellular β-crystallin aggregation. However, the pathogenesis of UVB-induced lens damage is still poorly understood.

The lens of the vertebrate eye is a unique organ which is avascular and contains only a single layer of epithelial cells on its anterior surface. This single layer of cells is the first region of the lens exposed to environmental insult, and is essential for maintaining homeostasis and transparency of the entire lens [7]. Multiple studies suggest that the lens epithelium is capable of communicating with underlying fiber cells [8] and direct damage to the lens epithelium results in cataract formation [9,10]. Several studies have shown that abnormal proliferation of lens epithelial cells at the equator and underneath the anterior lens capsule is induced by several risk factors, such as diabetes and UV light, leading to the development of cataract [11,12]. However, the gene expression changes and cellular processes in lens epithelial and fiber cells after UVB exposure are poorly understood. An important step in understanding UV-induced cataractogenesis is to identify biochemical and metabolic pathways that become altered during UVB exposure in lens epithelial cells.

In the present study, we have tried to identify gene expression differences between normal and UVB-exposed human lens epithelial (HLE) cells to gain a better understanding of the mechanism of action of UVB induced lens damage. We have focused on genes that encode extracellular proteins, especially growth factors and cytokines, since proteins secreted as a result of UVB stress would affect communication between the lens epithelium and underlying fiber cells, thus leading to pathological changes in the lens tissue. The data of this study provide an information resource relating to gene expression induced by UVB stress in HLE cells.

Our study has identified 18 secreted protein-coding genes that are upregulated more than twofold in UVB-exposed human lens epithelial cells. From these genes, we chose the gene products amphiregulin (*AREG*) and growth differentiation factor 15 (*GDF15*), and showed that they have stimulating activities on the rates of proliferation and protein synthesis of HLE cells.

## Methods

### Materials

Fetal bovine serum (FBS), Dulbecco's modified Eagle's medium (DME), MEM non-essential amino acids (NEAA), HEPES, penicillin-streptomycin and L-glutamine were obtained from Invitrogen Corp. (Carlsbad, CA). MEM Earle's medium without leucine (C-75240) was purchased from PromoCell (Heidelberg, Germany). Human recombinant epidermal growth factor (EGF) was from PeproTech (Rocky Hill, NJ). Human recombinant AREG (*E.coli*-derived Ser101-Lys198) and human GDF15 (CHO-derived Ala197-Ile308, with an N-terminal 6-His tag) were from R&D Systems (Minneapolis, MN). [Methyl-^3^H]-thymidine (NET027) and L-[4,5-^3^H(N)]-leucine (NET135H) were from PerkinElmer Inc. (Waltham, MA). Gelatin solution for coating was from Kurabo Industries (Osaka, Japan).

### Cell culture

The SV40 T-antigen-transformed human lens epithelial cell line, SRA01/04 [13] was provided by Dr. Nobuhiro Ibaraki (Department of Ophthalmology, Jichi Medical University, Tochigi, Japan). SRA01/04 cells expressed crystallin αA (*CRYAA*) mRNA; the mRNA level in SRA01/04 cells was lower than that in primary cultured human lens epithelial (HLE) cells but higher than that in HeLa cells. The data were consistent with the original report by Ibaraki et al. [13]. Cells were cultured in DME supplemented with 20% FBS, NEAA, HEPES, penicillin and streptomycin (complete medium) at 37 °C in a humidified atmosphere containing 5% CO_2_ [13]. Culture plates were coated for 30 min at 37 °C with gelatin solution before cell inoculation.

### UVB irradiation

Two UVB FL-15E 15W bulbs (Tozai Elec. Indus., Osaka, Japan) with peak emission at 313 nm were used. When cells were exposed to UVB, culture dishes were covered with a WG295 sharp cut filter (100 mm square; Shibuya Optical, Wako, Japan) to filter light below 295 nm. Under these conditions, cells received UV light of approximately 39% UVA, 50% UVB, and <1.5% UVC, with a total output of 0.345 mW/cm^2^, as monitored with a UVX radiometer (UVP, Upland, CA).

SRA01/04 cells were seeded at a density of 10^4^/cm^2^ in 60-mm or 100-mm dishes (BD Falcon, Oxnard, CA) and cultured for 2 days. Prior to irradiation, cells (95% confluence) were washed twice with warm phosphate-buffered saline (PBS), and then supplied with cold PBS. Cells were put on ice and then irradiated with various UVB-energy levels (0, 10, 30, 50, and 80 mJ/cm^2^). Time of irradiation was 29 to 232 s. After irradiation, the cells were cultured further for 12 h or 24 h in complete medium.

### Cell viability analysis

SRA01/04 cells were inoculated at 1.4×10^4^/well in a gelatin-coated 96-well plate and cultured for 12 h before UVB irradiation. The 96-well plate was covered with a black paper mask with a window opened to 4 neighboring wells whose cells were irradiated at 0, 10, 30, 50, or 80 mJ/cm^2^, and then cultured further for 12 h or 24 h. Viable cell numbers were assayed with a cell counting kit-F (Wako Pure Chem., Osaka, Japan) according to the manufacturer’s instructions. Cell viability was calculated by dividing the fluorescence unit of irradiated cells by that of non-irradiated cells.

### DNA microarray analysis

Total RNA was isolated using a commercially available kit (RNeasy Mini Kit; QIAGEN GmbH, Hilden, Germany). RNA was quantified by photometry at 260/280 nm, and the quality of the RNA was determined by the ratio of the 18S/28S ribosomal band intensities in an ethidium bromide-containing 1% agarose gel after electrophoresis. Preparation of sense cDNAs was performed using an Ambion® WT Expression Kit (Ambion, Austin, TX) and target hybridizations were performed using a Human Gene 1.0 ST Array which were composed of 28,869 separate probes (Affymetrix, Santa Clara, CA) according to the manufacturer’s instructions. Hybridization was performed for 17 h at 45 °C in a GeneChip® Hybridization Oven 640 (Affymetrix). After washing and staining in a GeneChip® Fluidics Station 450, hybridized cRNAs were detected using a GeneChip® Scanner 3000. The digitalized image data were processed using the GeneChip® Operating Software (GCOS) version 1.4. Since replicate assays were not performed, the signal intensities of selected genes that were upregulated or down-regulated by at least twofold compared to a sham-control group were extracted by a GeneSpring GX software package version 10.0 (Agilent Technologies, Santa Clara, CA). Ingenuity Pathway Analysis (IPA; Ingenuity Systems Inc., Redwood City, CA) was used as an additional method for evaluating the functional significance of the UVB-induced gene profiles.

### Real-time polymerase chain reaction (PCR)

Total RNAs were isolated from SRA01/04 cells or primary cultured HLE cells using a RNeasy Mini Kit or RNeasy Plus Mini Kit (QIAGEN). Real-time PCR analyses were performed using TaqMan Gene Expression Assays (Applied Biosystems, Carlsbad, CA: Hs00950668_m1 for *AREG*, Hs00171132_m1 for *GDF15*, and Hs99999905_m1 for glyceraldehyde-3-phosphate dehydrogenase [*GAPDH*]). Briefly, an aliquot (20–200 ng) of total RNA was reverse-transcribed into first-strand cDNA at 37 °C for 120 min using a High Capacity cDNA Reverse Transcription kit (Applied Biosystems) in a 20-µl reaction mixture according to the manufacturer’s instruction. Real-time PCR was performed with a Step One Real Time PCR System (Applied Biosystems). cDNA derived from 1.4 to 6.7 ng of total RNA was amplified with TaqMan Gene Expression Assays (FAM-MGB) and TaqMan Universal PCR Master Mix, No AmpErase UNG (Applied Biosystems) in the reaction volume of 10 µl according to the manufacturer’s instructions. Relative quantification of gene expression with real-time PCR data was calculated relative to *GAPDH*.

### Reverse-transcription PCR (RT–PCR)

RT–PCR was performed with a Thermal Cycler Dice TP600 (Takara Bio. Inc., Otsu, Japan) using a SuperScript One Step RT–PCR System with Platinum Taq (Invitrogen Corp., Carlsbad, CA) and total RNA (20–100 ng) in a 25-µl reaction mixture. The PCR settings were as follows: after the RT reaction at 55 °C for 30 min, the initial denaturation for 2 min at 94 °C was followed by 20 to 38 cycles of amplification cycle of 94 °C for 15 s, 60 °C for 30 s, and 68 °C for 30 s or 60 s. An endogenous control mRNA, β-actin (*ACTB*) mRNA, was also amplified. Aliquots (5–10 μl) of each RT–PCR product were electrophoresed on 2% agarose gels. Gels were stained with SYBR Green I (1:10,000 dilution) in 10 mM Tris-HCl (pH 8.0) for 15 min for quantification of RT–PCR products, and stained gels were scanned by Typhoon 9400 (GE Healthcare Bio-Sciences AB, Uppsala, Sweden) and the extent of band strength analyzed by ImageQuant™ TL7.0 software (GE Healthcare). The primer pairs for RT–PCR are listed in Table 1.

**Table 1:** Primers used for RT–PCR analyses.

**Primer names**	**Nucleotide sequences**	**GenBank accession number**	**Product size**
*AREG* forward	5′-TGCTGGATTGGACCTCAATG-3′	NM_001657	163 bp
*AREG* reverse	5′-TCCCGAGGACGGTTCACTAC-3′	** **	** **
*GDF15* forward	5′-CGGTGAATGGCTCTCAGATG-3′	NM_004864	167 bp
*GDF15* reverse	5′-CAGGTCCTCGTAGCGTTTCC-3′	** **	** **
*CRYAA* forward	5′-TTTTGAGTATGACCTGCTGCC-3′	NM_000394	252 bp
*CRYAA* reverse	5′-TGGAACTCACGGGAAATGTAG-3′	** **	** **
*EGFR* forward	5′-CAGCGCTACCTTGTCATTCA-3′	NM_005228	247 bp
*EGFR* reverse	5′-AGCTTTGCAGCCCATTTCTA-3′	** **	** **
*TGFBR1* forward	5′-AAATTGCTCGACGATGTTCC-3′	NM_004612	309 bp
*TGFBR1* reverse	5′-GGAGAGTTCAGGCAAAGCTG-3′	** **	** **
*TGFBR2* forward	5′-TTTTCCACCTGTGACAACCA-3′	NM_001024847	342 bp
*TGFBR2* reverse	5′-GCTGATGCCTGTCACTTGAA-3′	** **	** **
*EGF* forward	5′-AGATGGGAAAACGTGTCTGG-3′	NM_001963	246 bp
*EGF* reverse	5′-CACTGACATGTGGCATCCTC-3′	** **	** **
*ACTB* forward	5′-CATCGAGCACGGCATCGTC-3′	NM_001101	507 bp
*ACTB* reverse	5′-CTCTTCTCCAGGGAGGAGC-3′	** **	** **

### ELISA

SRA01/04 cells were inoculated in duplicate at 6×10^5^/plate in 60-mm plates and cultured to become confluent after 2 days. UVB irradiation was performed as described above. Conditioned media were collected at 12 h and 24 h, and centrifuged at 1,500× g for 10 min, and the supernatants stored at −20 °C until ELISA assays were conducted. ELISA assays were performed using a human amphiregulin DuoSet ELISA Development System and a human GDF15 Quantikine ELISA kit (R&D Systems. Inc., Minneapolis, MN) in triplicate wells according to the manufacturer’s instructions.

### Primary culture of human lens epithelial (HLE) cells

Primary cultured HLE cells were prepared from capsular flaps removed surgically in intraocular lens implantation. The capsular flap was split in half, and each half was placed in the center of wells in a 35-mm plate with a small amount of complete medium. The tissues were allowed to stand for 5 min and then supplemented with 1.5 ml of complete medium, and incubated at 37 °C in a humidified atmosphere containing 5% CO_2_. The HLE cells grew beyond the capsular edge 3–5 days after the beginning of cultivation and expanded actively to the periphery of the culture well. Cells which had been cultured for 2 weeks were used for experiments. Lens capsules used for primary HLE cultures (A – E) were donated from senile cataract patients. Their ages and types of cataract diagnosed by the WHO grading system [14] were as follows, respectively; A: 76 and cortical (grade2), B: 52 and cortical (grade1), posterior subcapsular cataract (PSC) (grade1), C: 81 and PSC (grade3), D: 54 and cortical (grade1), E: 79 and nuclear (grade1), cortical (grade3). Studies were performed with approval from the Kanazawa Medical University ethics committee. Informed consent was obtained from each participant before the study. All procedures conformed to the tenets of the Declaration of Helsinki.

### ^3^H-thymidine and ^3^H-leucine uptake

SRA01/04 cells were inoculated at 6×10^4^/well in a gelatin-coated 24-well plate, and cultured for 4 h to become attached. Medium was replaced by 1 ml of DME (for ^3^H-thymidine uptake) or MEM Earle's medium containing 40 µM L-leucine (for ^3^H-leucine uptake) supplemented with 0.2% FBS and cultured for 24 h. After the incubation, the medium was replaced and recombinant AREG, GDF15, or epidermal growth factor (EGF) was added to the cultures. Then 5 µl of ^3^H-thymidine (1.48 kBq/µl) in 0.2 mM thymidine or 5 µl of ^3^H-leucine (1.85 kBq/µl) was added to the wells and the cells were incubated for 5 h. Acid-insoluble ^3^H-radioactivities in the wells were measured by liquid scintillation counting [15].

### Statistical analysis

Values were expressed as the mean±SD of at least three independent experiments. Statistical significance was determined by performing the Student’s *t*-test. p values less than 0.05 were considered statistically significant.

## Results

### Effect of UVB exposure on the viability of SRA01/04 cells

**Figure 1:**
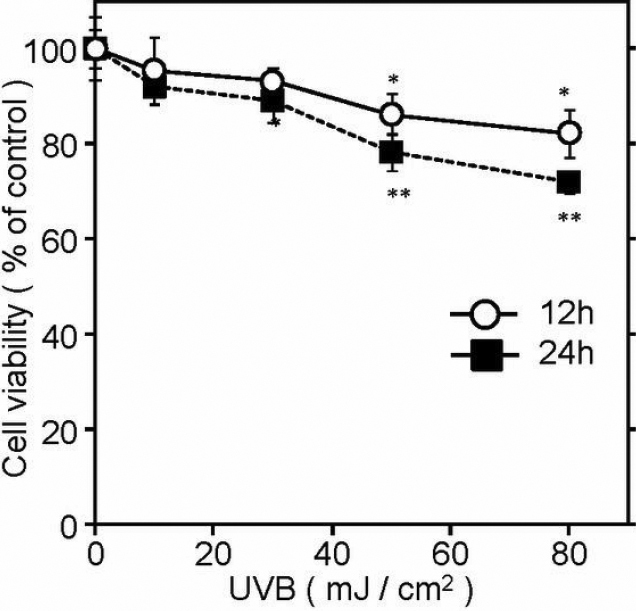
Effect of UVB exposure on the viability of SRA01/04 cells. SRA01/04 cells were irradiated at indicated energies of UVB and cultured further for 12 h or 24 h, and viable cell numbers assayed (n=4). Cell viability is shown as % of control (sham-irradiated culture). Essentially the same results were obtained by three independent experiments and representative data are shown. **p<0.01; *p<0.05, compared to controls.

We first checked the effect of UVB irradiation on SRA01/04 cell viability as described under Methods. After UVB irradiation at various energy levels, we assayed cell numbers at time points of 12 h and 24 h since these are the times at which apoptotic processes have peaked and DNA repair processes have substantially finished [16,17]. As shown in Figure 1, UVB exposure produced a cytotoxic effect on the cells in an energy-dependent manner. UVB irradiation at 30 mJ/cm^2^ slightly reduced cell viability to 93% at 12 h and to 89% at 24 h. Even when the irradiation energy was increased to 50 mJ/cm^2^, the cell viability was kept at 86% and 78% at 12 h and 24 h, respectively, under our experimental conditions. The irradiation condition of 30 mJ/cm^2^ was thus adopted for DNA microarray analysis.

### Affymetrix microarray analysis for the genes upregulated by UVB exposure

To examine effects of UVB exposure on overall gene expression, we performed a DNA microarray analysis of gene expression in UVB (30 mJ/cm^2^)-exposed SRA01/04 cells at time points of 12 h and 24 h. The majority (97.7%–99.4%) of signal intensities of UVB-irradiated cells were essentially unchanged (between 0.5 and 2.0 fold) as compared with that of control non-irradiated cells (data not shown). At the 12 h time point, we detected 61 genes that were upregulated more than 2 fold by UVB exposure, and 580 genes that were down-regulated less than 0.5 fold by UVB exposure. At the time point 24 h after irradiation, we detected 44 genes that were upregulated more than twofold, and 116 genes that were down-regulated less than 0.5 fold. Genes upregulated at 12 h or 24 h were combined, resulting in a pool of 94 genes. The probable biologic functions of the genes were associated with apoptosis, survival, cellular growth and proliferation, cancer, and DNA synthesis (data not shown). Genes that were upregulated by UVB exposure were thought to play important roles in the cell response to UVB stress.

Proteins secreted as a result of UVB stress could affect lens cell growth and metabolism, thus leading to pathological changes of lens tissue. We therefore focused on genes which encode extracellular proteins, especially growth factors and cytokines. Table 2 shows 18 secreted protein genes that were upregulated more than twofold at either or both time points of 12 h and 24 h post irradiation. We decided to focus on *AREG* and *GDF15* since these proteins have not been studied before with regard to UVB, and their induced expression extended to 24 h. Pathological changes of the human lens as a result of UVB exposure are thought to be due to long-term, chronic effects.

**Table 2:** UVB-irradiation induced changes in gene expression whose products located in extracellular space.

** **	** **	**Fold change**	
**Gene**	**Gene description**	**12 h**	**24 h**
*ESM1*	endothelial cell-specific molecule 1	1.80	4.86
*SERPINB2*	serpin peptidase inhibitor, cladeB, member 2	1.80	4.22
*IL1B*	interleukin 1β	1.85	4.14
*AREG*	amphiregulin	3.20	3.94
*LAMB3*	laminin, β3	1.19	3.56
*GDF15*	growth differentiation factor 15	1.89	3.42
*PTX3*	pentraxin-related gene, rapidly induced by IL-1β	2.36	2.90
*TFPI2*	tissue factor pathway inhibitor 2	1.89	2.55
*TNFSF4*	tumor necrosis factor (ligand) superfamily, member 4	1.10	2.36
*FRZB*	frizzled-related protein	1.94	2.30
*EDN1*	endothelin 1	0.87	2.27
*TAGLN3*	transgelin 3	2.28	2.11
*CCL26*	chemokine (C-C motif) ligand 26	1.18	2.00
*HBEGF*	heparin-binding EGF-like growth factor	2.92	1.94
*IL6*	interleukin 6 (interferon, β2)	2.51	1.73
*STC1*	stanniocalcin 1	2.38	1.60
*FST*	follistatin	2.42	1.53
*TGFB3*	transforming growth factor, β3	2.26	1.19

Genes that gave the fold increases of signal intensity more than 2.0 at 12 h and/or 24 h after UVB irradiation are shown.

### RT–PCR and real-time PCR analyses of *AREG* and *GDF15* expression

To confirm the observed upregulation of *AREG* and *GDF15* as a result of UVB exposure, RT–PCR was conducted with the original RNA samples used for the microarray experiments. *GAPDH* or *ACTB* were used as endogenous controls for real-time PCR and RT–PCR, since the variations of raw signals of *GAPDH* and *ACTB* were within 2% and 6%–10%, respectively, between UVB exposed and unexposed cells in our microarray data. The *AREG* mRNA levels in the 30 mJ/cm^2^-exposed SRA01/04 cells were increased 4.1 and 4.5 fold at 12 h and 24 h, respectively, compared with those in the control unexposed cells (data not shown). The *GDF15* mRNA levels in the 30 mJ/cm^2^-exposed SRA01/04 cells were also increased 4.6 and 5.2 fold at 12 h and 24 h, respectively (data not shown).

**Figure 2:**
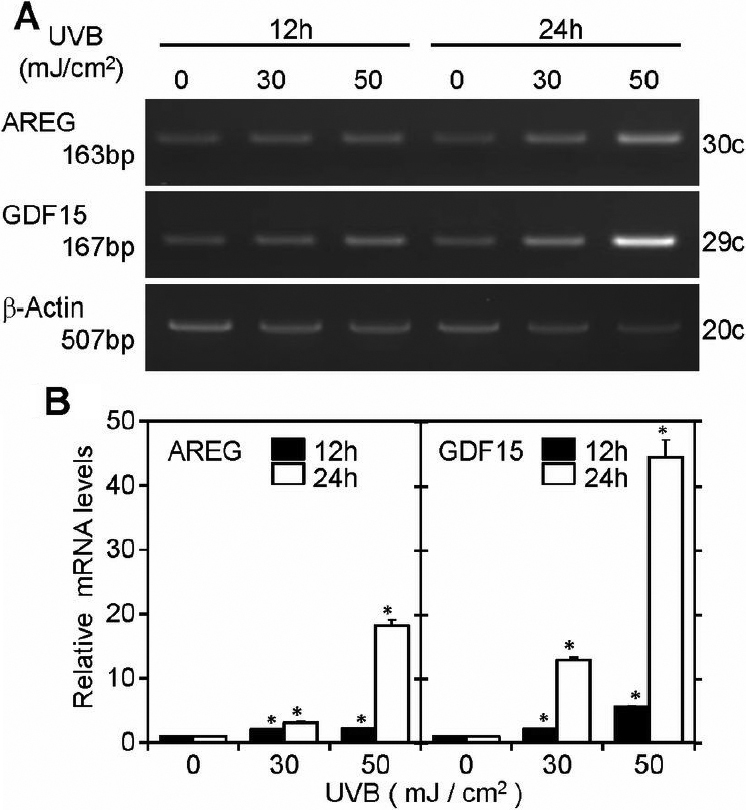
RT–PCR and real-time PCR analysis of *AREG* and *GDF15* expression in UVB-irradiated SRA01/04 cells. SRA01/04 cells were exposed at 0, 30, and 50 mJ/cm^2^ UVB and total RNAs were extracted 12 h and 24 h later. Relative mRNA abundance of *AREG* and *GDF15* was examined using RT–PCR (**A**) and real-time PCR (**B**). **A**: RT–PCR products of *AREG*, *GDF15*, and β-actin (*ACTB*) mRNAs. The RNA amounts and PCR cycle numbers were 100 ng and 30 cycles (*AREG*), 100 ng and 29 cycles (*GDF15*), and 100 ng and 20 cycles (*ACTB*). Aliquots (10 μl) of each RT–PCR product were electrophoresed on 2% agarose gels containing ethidium bromide. **B**: Relative mRNA levels of *AREG* (left). Relative mRNA levels of *GDF15* (right). Values were normalized with *GAPDH* mRNA, and compared to values of controls (sham-irradiated cells). Essentially the same results were obtained with three independent experiments, and representative data are shown. *p<0.001, compared to controls.

Next, we prepared different batches of RNA samples from cells which had been exposed at 0, 30 and 50 mJ/cm^2^ UVB and determined the reproducibility of the experiments (Figure 2). As shown as Figure 2A, RT–PCR bands were observed at each of the predicted sizes. New batches of RNA samples were examined for *AREG* and *GDF15* expression by real-time PCR. *AREG* expression in 30 and 50 mJ/cm^2^-UVB exposed cells was upregulated 2.1 and 2.3 fold, respectively, at 12 h, and was further upregulated at 24 h to 3.1 and 18.2 fold at 30 and 50 mJ/cm^2^, respectively (Figure 2B). *GDF15* expression in 30 and 50 mJ/cm^2^-UVB exposed cells was upregulated to 2.1 and 5.6 fold, respectively, at 12 h, and was dramatically upregulated at 24 h to 12.4 and 44.4 fold at 30 and 50 mJ/cm^2^, respectively (Figure 2B). Fragments amplified by RT–PCR were represented clearly in heavy bands at 24 h after 50 mJ/cm^2^ exposure as shown in Figure 2A. This extensively high expression led us to attempt detection of proteins in the conditioned media of HLE cells which had been subjected to UVB irradiation.

### AREG and GDF15 protein levels in conditioned media of UVB-exposed cells

**Figure 3:**
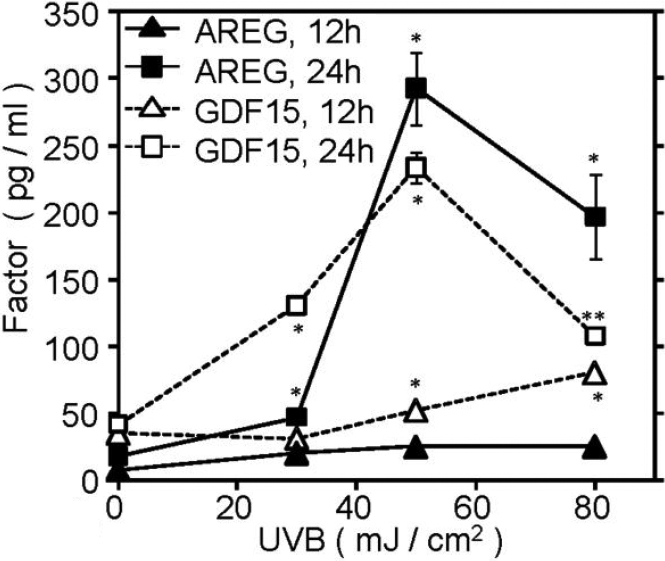
AREG and GDF 15 protein level in conditioned media of UVB-exposed cells. SRA01/04 cells were irradiated at indicated energies of UVB. The conditioned media were collected after 12 h and 24 h, and were examined for AREG and GDF15 ELISA assays. Values are expressed as the mean±average deviation in biologic duplicate determinations. Solid triangle and square were AREG protein level at 12 h and 24 h, respectively. Open triangle and square were GDF15 protein level at 12 h and 24 h, respectively. Essentially the same results were obtained with three independent experiments, and representative data are shown. **p<0.01; *p<0.05, compared to control conditioned media (sham-irradiated culture).

We next examined protein levels of AREG and GDF15 in conditioned media of SRA01/04 cells which had been subjected to UVB irradiation. We prepared conditioned media of cells which had been irradiated at various UVB-energy levels and analyzed by ELISA assays (Figure 3). The AREG protein levels significantly increased at all UVB-energy points at 24 h, whereas the immuno-reactive AREG was scarcely detectable at 12 h after UVB exposure. The highest concentration of AREG was observed at 50 mJ/cm^2^ (293 pg/ml, 36.6 pg/10^5^ cells). The value of AREG at 80 mJ/cm^2^ was lower than that of 50 mJ/cm^2^, probably because of decreased cell viability as shown in Figure 1. Immuno-reactive GDF15 levels also increased in conditioned media collected at 12 h and 24 h in a similar pattern to AREG (a maximum at 50 mJ/cm^2^ of 233 pg/ml, 29.1 pg/10^5^ cells). Thus, upregulated protein secretions of AREG and GDF15 were coincident with upregulation of their mRNA levels.

### Expression of *AREG* and *GDF15* genes in UVB-exposed primary cultured HLE cells

**Figure 4:**
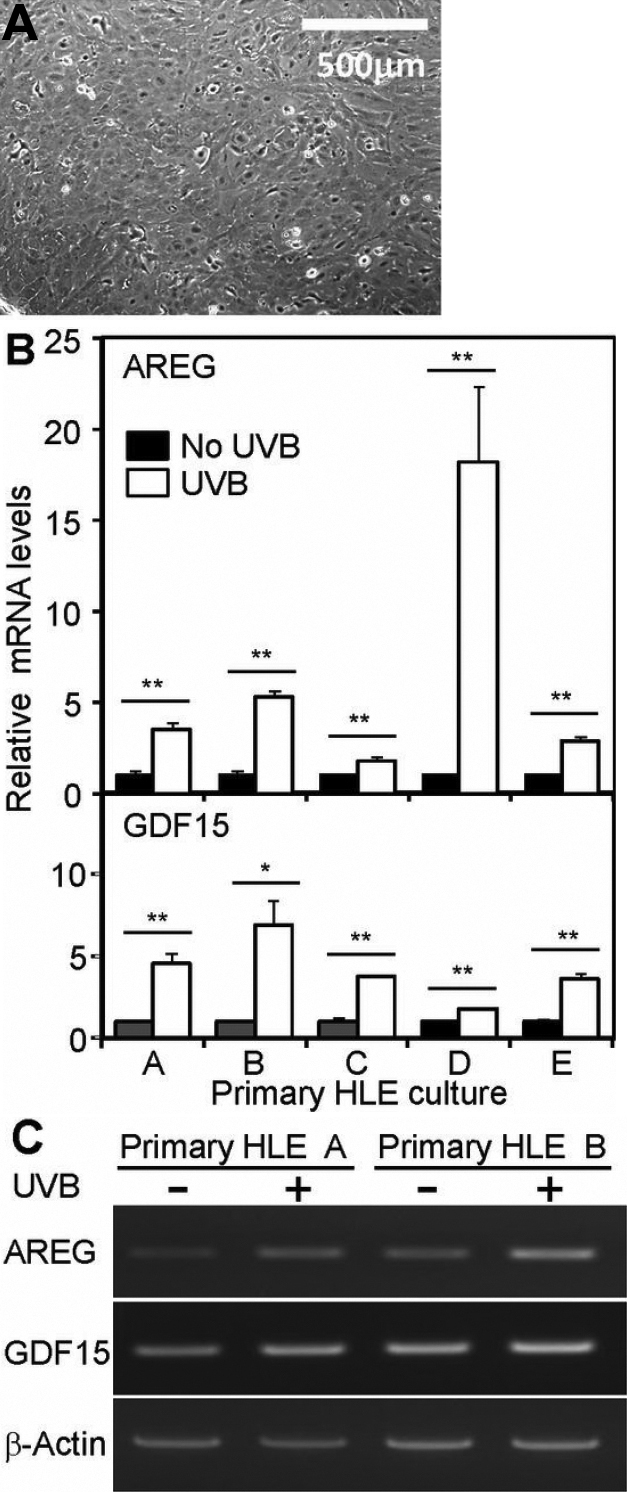
Morphology of primary cultured HLE cells and expression of *AREG* and *GDF15* in UVB-exposed primary cultured HLE cells. The capsular flaps were obtained from 5 patients (A–E) and split in half, and halves were cultured for 2 weeks. **A**: A typical phase contrast micrograph of primary culltured HLE cells after a 2-week culture is shown. Note that almost all the cells outgrown from the capsules had small, polygonal shapes. One of doublet cultures was irradiated at 50 mJ/cm^2^ and another culture was subjected to sham operation. Total RNAs were extracted at 24 h after UVB exposure and relative mRNA levels of *AREG* and *GDF15* were analyzed using real-time PCR (**B**) and RT–PCR (**C**). **B**: Relative mRNA levels of *AREG* (upper). Relative mRNA levels of *GDF15* (lower). Values were normalized with *GAPDH* mRNA. The respective values of UVB-exposed culture are compared to values of the corresponding sham-operated cultures (No UVB). *p<0.05, **p<0.01, compared to controls (No UVB). **C**: RT–PCR products of RNAs from cultures for patient A and B. Aliquots (10 μl) of each RT–PCR product for *AREG* (20 ng, 38 cycles), *GDF15* (20 ng, 33 cycles) and β-actin (*ACTB*; 20 ng, 24 cycles) were electrophoresed on 2% agarose gels containing ethidium bromide.

To further confirm upregulation of *AREG* and *GDF15* in UVB-exposed human lens epithelium, we prepared doublet wells of primary HLE cell cultures derived from two halves of capsular flaps surgically removed from 5 patients who had given informed consent. It was thus possible to compare mRNA expressions in UVB-exposed and unexposed cells. It has been reported that there is only one cell type, lens epithelial cells, in the lens capsule [18]. As shown in Figure 4A, almost all the cells outgrown from the capsules had small, polygonal shapes, which are the typical morphologies of epithelial cells, as reported previously [18].

We first confirmed that RNA samples from each half flap culture gave the same expression levels of *AREG* and *GDF15* relative to *GAPDH* expression levels (data not shown). Next, primary cultured HLE cells were irradiated at 50 mJ/cm^2^ and further incubated for 24 h. Morphological changes of primary HLE cells after UVB exposure were not apparent, as observed under phase contrast microscopy. Total RNAs were isolated from both UVB-exposed and non-exposed cultures and analyzed for *AREG* and *GDF15* expression using real-time PCR. As shown in Figure 4B, upper panel, *AREG* mRNA levels were significantly upregulated (1.8–18.2 fold for the 5 patients A–E) in the UVB-exposed cultures compared with the corresponding control cultures. *GDF15* mRNA levels were also significantly upregulated (1.7–6.8 fold for the 5 patients A–E) in the UVB-exposed cultures compared with the corresponding control cultures (Figure 4B lower panel). The basal *AREG* mRNA levels in no-UVB cultures were 1.0 (A), 2.0 (B), 4.1 (C), 2.5 (D), and 11.9 (E), when the mRNA levels were related to the value of culture A. The basal *GDF15* mRNA levels in no-UVB cultures were 1.0 (A), 2.7 (B), 2.1 (C), 4.1 (D), and 5.7 (E), when the mRNA levels were related to the value of culture A. Since the number of the examined samples was small, we could not find any relationship between cataract type/severity of lens opacities and either the basal levels or the extent of UVB-induced upregulation of *AREG* and *GDF15* mRNAs. Figure 4C shows RT–PCR products of cultures for patients A and B. The results were consistent with those obtained by real-time PCR analysis. These results indicated that primary HLE cells responded to UVB exposure in the same way as observed for SRA01/04 cells in regards to *AREG* and *GDF15* expression.

### Effects of recombinant AREG and GDF15 proteins on SRA01/04 cell proliferation and protein synthesis

**Figure 5:**
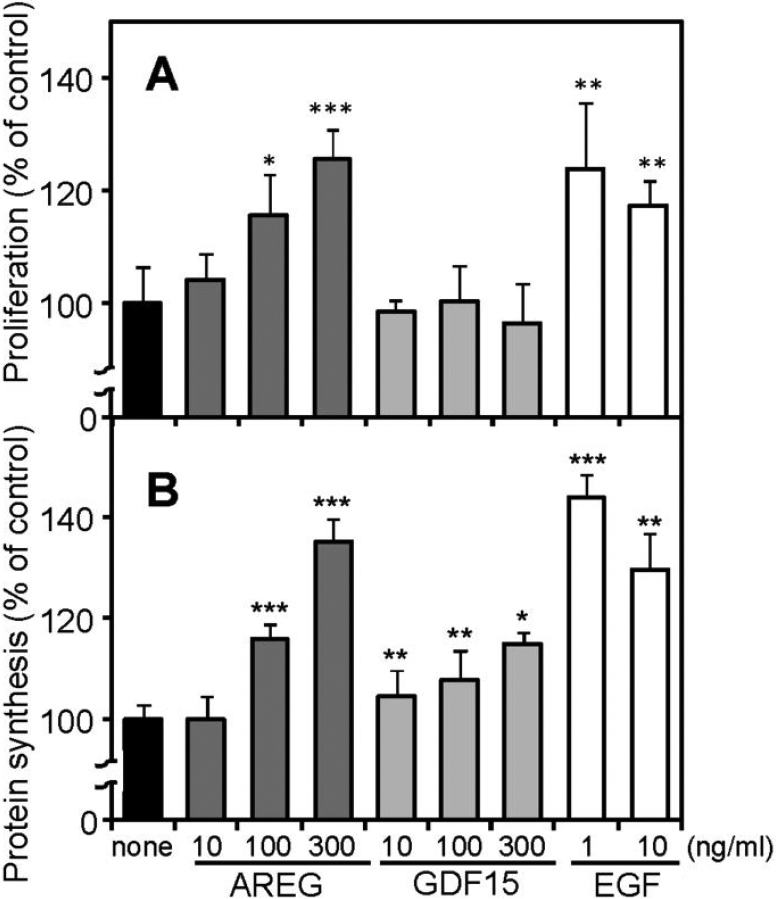
Effects of recombinant AREG and GDF15 proteins on cell proliferation and protein synthesis of SRA01/04 cells. Serum starved SRA01/04 cells were incubated with recombinant AREG, GDF15, or EGF at the indicated concentrations and examined ^3^H-thymidine (**A**) or ^3^H-leucine (**B**) uptake as described in Methods. Values are expressed as the mean±SD (n=4~5) and presented as % of control (none). Essentially the same results were obtained by three times and representative data are shown. ***p<0.001; **p<0.01; *p<0.05, compared to controls (none).

To examine whether AREG and GDF15 secreted as a result of UVB exposure have any biologic effects on HLE cells, we cultured SRA01/04 cells in the presence of recombinant AREG and GDF15 proteins, and examined ^3^H-thymidine incorporation to measure proliferation and ^3^H-leucine incorporation to measure protein synthesis. EGF was used as a positive control. As shown in Figure 5A, AREG significantly stimulated ^3^H-thymidine incorporation (116% at 100 ng/ml and 126% at 300 ng/ml) as did the positive control, EGF. AREG also significantly stimulated ^3^H-leucine uptake (116% at 100 ng/ml and 135% at 300 ng/ml) (Figure 5B). The effective dose of AREG, however, was about 100 times higher than that of EGF for both ^3^H-thymidine and ^3^H-leucine incorporation.

Recombinant GDF15 did not stimulate ^3^H-thymidine incorporation at any concentration tested (Figure 5A), but significantly stimulated ^3^H-leucine uptake at 10–300 ng/ml (108%–115%; Figure 5B). These results indicated that AREG and GDF15 produced in response to UVB exposure affected the growth and protein synthesis of lens epithelial cells, suggesting their autocrine and paracrine roles in pathological changes of lens tissue during long-term UVB exposure.

### Expression of mRNAs for EGF receptor (*EGFR*), *EGF*, and TGFβ receptors (*TGFBR1* and *TGFBR2*) in primary HLE and SRA01/04 cells

**Figure 6:**
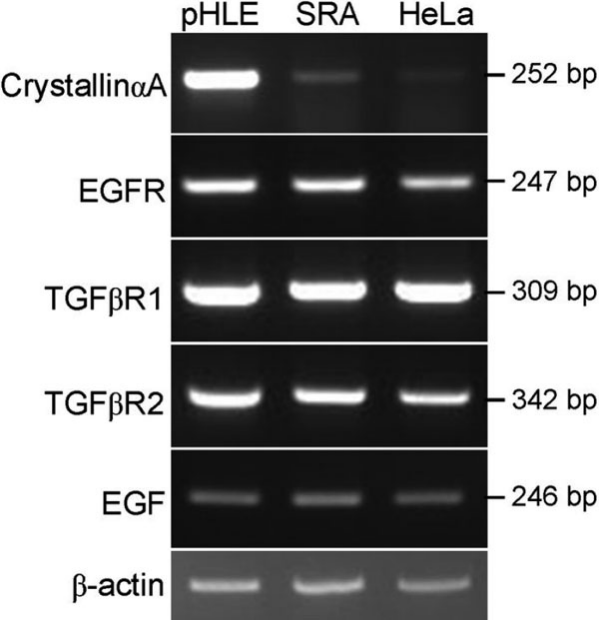
Expression of mRNAs for crystallin αA, EGF receptor (*ERBB-1*), TGFβ receptors, and EGF in primary cultured HLE cells (pHLE) and SRA01/04 cells (SRA). Total RNAs were extracted from primary cultured HLE and SRA01/04 cells, and were analyzed by RT–PCR using the primers listed in Table 1. RNA from HeLa cells was also analyzed as control. Aliquots (10 μl) of each RT–PCR product for crystallin αA (*CRYAA*; 100 ng, 35 cycles), EGF receptor (*EGFR*; 50 ng, 35 cycles), TGFβ receptor-1 (*TGFBR1*; 50 ng, 35 cycles), TGFβ receptor-2 (*TGFBR2*; 50 ng, 35 cycles), *EGF* (50 ng, 35 cycles) and β-actin (*ACTB*; 50 ng, 23 cycles) were electrophoresed on 2% agarose gels containing ethidium bromide.

As shown in Figure 5, AREG and GDF15 proteins stimulated the growth and/or protein synthesis of SRA01/04 cells. We thus confirmed the expression of EGF receptor (ERBB1) and TGFβ receptors in primary cultured HLE and SRA01/04 cells. RT–PCR analysis demonstrated that both cells express EGF receptor mRNA along with *EGF* mRNA (Figure 6). The EGF receptor mRNA levels were similar in primary cultured HLE cells and SRA01/04 cells. The *EGF* mRNA level in primary cultured HLE cells was lower than that in SRA01/04 cells. Primary cultured HLE cells and SRA01/04 cells also express mRNAs for TGFβ receptor-1 and −2, and the signal intensities of both mRNAs were similar in primary cultured HLE and SRA01/04 cells (Figure 6).

## Discussion

The effect of UVB radiation on animal cells has been well investigated in dermal keratinocytes and fibroblasts and shown to produce severe DNA damage [19]. UVB radiation also produces reactive oxygen species (ROS). ROS activates the transcription factor NF-κB, which induces expression of pro-inflammatory cytokines (IL-1/6, TNF-α). ROS also inhibit the enzyme protein-tyrosine phosphatase-κ leading to activation of receptor tyrosine kinases and intracellular signaling, which activate the nuclear transcription complex AP-1. AP-1 increases transcription of matrix metalloproteases and decreases expression of the procollagen I and III genes, with a final consequence of reduced extracellular matrix formation [20]. However, the gene expression, cellular processes and intercellular communication that lead to cataracts in UVB-exposed lens tissues are poorly understood.

In this work, we investigated the effect of UVB irradiation on gene expression of HLE cells using a human lens epithelial cell line, SRA01/04. Shui et al. [21] reported that UVB irradiation of SRA01/04 cells at 10 mJ/cm^2^ produced significant TUNEL positive cells at 12 h (18.6±14.9%) and 24 h (32.5±26.7%), while only 4.3±3.6% of cells were TUNEL positive in non-irradiated cultures. Under our conditions, nearly 90% of UVB-irradiated cells were viable 24 h after irradiation at 30 mJ/cm^2^ (Figure 1). Thus we used 30 mJ/cm^2^ for DNA microarray analysis.

DNA microarray analysis identified 61 and 44 genes upregulated by UVB exposure at 12 h and 24 h time points, respectively (the data were not shown). The genes encoded a variety of proteins such as transcription factors, DNA damage-related proteins, and stress response proteins. We focused our attention on extracellular proteins (Table 2), since such secreted proteins would have roles in communicating between lens epithelial cells and underlying fiber cells, and thus might contribute to the pathogenesis of UVB-induced cataractogenesis. Our finding that the pro-inflammatory cytokines IL-1β and IL-6 were upregulated by UVB irradiation in HLE cells is consistent with previous reports on photoaging of skin [19].

In our study, *AREG*, which has not been investigated previously in HLE cells, was prominently upregulated by UVB exposure (Table 2). We thus focused on *AREG* and examined its expression and function in HLE cells. AREG is one of six mammalian ligands that bind EGF receptor [22]. AREG protein is synthesized as a pro-AREG trans-membrane glycoprotein, and is sequentially cleaved within the ectodomain to be released as a soluble AREG form [23,24]. RT–PCR and real-time PCR analysis confirmed upregulation of *AREG* in UVB-exposed SRA01/04 cells (Figure 2), and its protein levels were also significantly increased at 24 h, while it was scarcely detectable at 12 h (Figure 3). To confirm the upregulation of *AREG* in human lens epithelium, we examined UVB-induced expression of *AREG* in UVB exposed primary cultured HLE cells prepared from surgically removed human lens epithelium, and confirmed the UVB induced expression (Figure 4). We also found that AREG significantly stimulated ^3^H-thymidine and ^3^H-leucine uptake in SRA01/04 cells as did the positive control, EGF (Figure 5). These results indicated that AREG, which was produced in response to UVB exposure, can affect the growth and protein synthesis of HLE cells. The effective dose of AREG shown in Figure 5 was about 100 times higher than that of EGF. The lower effective dose may be due to the usage of recombinant AREG protein for the experiments. Thompson et al. [25] reported that *E. Coli-*derived AREG protein has lower affinity for EGFR than EGF. Naturally occurring AREG may have an effect on EGFR at a lower dose than recombinant proteins. The peak concentration of AREG (293 pg/ml) in SRA01/04 cell conditioned media (Figure 3) was lower than the effective dose of EGF (1 ng/ml). AREG and other factors would be secreted into a narrow space between lens epithelial cells and underlying fiber cells, and their local concentrations may be much higher than that in the conditioned media. Further, unlike EGF, AREG has a heparin binding domain [22] and can be stored and accumulated on extracellular matrix to further increase its local concentration. Thus, AREG may have autocrine and paracrine roles in the long term, chronic pathological changes occurring in lens tissues during UVB induced cataractogenesis.

RT–PCR analysis demonstrated that SRA01/04 and primary cultured HLE cells express EGF receptor along with EGF (Figure 6). Since EGF and AREG share the same receptor, AREG could modulate or disturb the EGF effects which may be important for homeostasis of lens tissues. The *EGF* mRNA level in primary cultured HLE cells was lower than that in SRA01/04 cells (Figure 6), suggesting that AREG can exert its effects on lens epithelial cells at a concentration lower than the effective concentrations to SRA01/04 cells. Members of the EGF family stimulate the EGF receptor to varying extents and in different manners [22,26]. While EGF activates EGF receptors in order for it to be internalized and degraded, AREG activates receptors in order for it to be accumulated near the cell surface [27]. AREG might activate other signaling pathways leading to a variety of cellular responses in addition to cell proliferation and protein synthesis via the EGF receptor in HLE cells. In fact, AREG has been reported to act as an anti-apoptotic factor, which was induced in response to liver cell damage [28,29]. We also detected the upregulation of another EGF-family member, heparin-binding EGF-like growth factor (*HB-EGF*), by UVB exposure of SRA01/04 cells (Table 2). Expression of AREG and HB-EGF has also been reported in a skin wound healing model [30]. Rittie et al. [31] reported that treatment of human skin with all-trans retinoic acid which caused an epidermal hyperplasia, increased mRNA and protein levels of AREG and HB-EGF. These observations suggest that simultaneous expression of AREG and HB-EGF might be common in stressed epithelial cells. The dual expression may cross-induce and co-operate with each other in epithelial cells in response to stress.

In this study, we also identified upregulation of *GDF15* by UVB irradiation in SRA01/04 cells and primary cultured HLE cells at both the mRNA and protein levels (Figure 2, Figure 3, Figure 4). This is also the first observation that *GDF15* is upregulated in HLE cells in response to UVB exposure. GDF15, a member of the TGFβ superfamily, is also known as MIC-1, PDF, PLAB, and NAG-1, and has a role in regulating inflammatory and apoptosis pathways during tissue injury and in certain diseases [32-35]. Recombinant GDF15 was not found to stimulate ^3^H-thymidine incorporation in SRA01/04 cells at any concentration tested, but it did significantly stimulate ^3^H-leucine uptake (Figure 5), indicating that GDF15 that is produced in response to UVB exposure can affect protein synthesis of HLE cells. RT–PCR analysis confirmed the expression of mRNAs for TGFβ receptor-1 and -2 (Figure 6). *GDF15* has been reported to be induced by H_2_O_2_ in human adipocytes [36], human lung epithelial cells [37], and human macrophages [38]. Recently, Akiyama et al. [39] demonstrated that *GDF15* is upregulated by blue or near-UV light in cultured normal human dermal fibroblasts. There have been several reports that GDF15 protein inhibits cell proliferation, similar to TGFβ; conditioned medium collected from GDF15-overexpressing cancer cells suppressed tumor cell growth through the TGFβ signaling pathway [40]. It has also been reported that GDF15 inhibits proliferation of primitive hematopoietic progenitors [41]. Our study showed that GDF15 can affect protein synthesis in HLE cells, but it might also be able to activate other signaling pathways via TGFβ receptors. It has been reported that GDF15 antagonizes the hypertrophic response and loss of ventricular performance, and protects cardiomyocytes from apoptosis during simulated ischemia/reperfusion as an autocrine factor [42,43]. These observations suggest that GDF15 might have a role in protecting HLE cells and/or fiber cells against UVB stress.

In conclusion, the present study has offered a glimpse of the variety of UVB-induced global gene expression changes occurring in HLE cells, and revealed *AREG* and *GDF15* as prominent upregulated genes produced by UVB exposure. AREG and GDF15 are able to modify growth and protein synthesis of lens epithelium, and can probably affect the metabolism of underlying fiber cells in a paracrine manner, and thus may contribute to pathological changes in UVB-induced cataractogenesis. In lens homeostasis and UVB-induced catalactogenesis, interaction between epithelial and fiber cells may be essential, and effects of AREG and GDF15 on fiber cells are quite important. To clarify the roles of AREG and GDF15, and other upregulated gene products in lens homeostasis and UVB-induced catalactogenesis, we are planning to do knockdown and overexpression approaches in vivo using animal models in a future study. Although additional studies are needed to better clarify the significance of *AREG* and *GDF15*, and other upregulated genes in UVB-exposed HLE cells, the present findings have revealed new regulatory features in lens homeostasis and UVB-induced cataractogenesis. AREG and GDF15, and other upregulated factors may additively or synergistically affect the metabolism of lens epithelial and fiber cells, leading to derangement of lens homeostasis. The results obtained in this study may provide new clues for clarifying the pathogenesis of cataracts and other lens-related diseases, and for developing preventive measures against them.

## References

[1] Kerr JB McElroy CT Evidence for large upward trends of ultraviolet-B radiation linked to ozone depletion. Science 1993 262 1032 4 17782050 10.1126/science.262.5136.1032

[2] Foster A Resnikoff S The impact of Vision 2020 on global blindness. Eye 2005 19 1133 5 16304595 10.1038/sj.eye.6701973

[3] Bochow TW West SK Azar A Munoz B Sommer A Taylor HR Ultraviolet light exposure and risk of posterior subcapsular cataracts. Arch Ophthalmol 1989 107 369 72 2923558 10.1001/archopht.1989.01070010379027

[4] McCarty CA Taylor HR Recent developments in vision research: light damage in cataract. Invest Ophthalmol Vis Sci 1996 37 1720 3 8759338

[5] Wickert H Zaar K Grauer A John M Zimmermann M Gillardon F Differential induction of proto-oncogene expression and cell death in ocular tissues following ultraviolet irradiation of the rat eye. Br J Ophthalmol 1999 83 225 30 10396203 10.1136/bjo.83.2.225 PMC1722944

[6] Michael R Vrensen G van Marle J Lofgren S Soderberg PG Repair in the rat lens after threshold ultraviolet radiation injury. Invest Ophthalmol Vis Sci 2000 41 204 12 10634622

[7] Piatigorsky J Lens differentiation in vertebrates. A review of cellular and molecular features. Differentiation 1981 19 134 53 7030840 10.1111/j.1432-0436.1981.tb01141.x

[8] Rae JL Bartling C Rae J Mathias RT Dye transfer between cells of the lens. J Membr Biol 1996 150 89 103 8699483 10.1007/s002329900033

[9] Hightower KR The role of the lens epithelium in development of UV cataract. Curr Eye Res 1995 14 71 8 7720407 10.3109/02713689508999916

[10] Spector A Oxidative stress-induced cataract: mechanism of action. FASEB J 1995 9 1173 82 7672510

[11] Marcantonio JM Rakic JM Vrensen GF Duncan G Lens cell populations studied in human donor capsular bags with implanted intraocular lenses. Invest Ophthalmol Vis Sci 2000 41 1130 41 10752951

[12] Saxby L Rosen E Boulton M Lens epithelial cell proliferation, migration, and metaplasia following capsulorhexis. Br J Ophthalmol 1998 82 945 52 9828783 10.1136/bjo.82.8.945 PMC1722713

[13] Ibaraki N Chen SC Lin LR Okamoto H Pipas JM Reddy VN Human lens epithelial cell line. Exp Eye Res 1998 67 577 85 9878220 10.1006/exer.1998.0551

[14] Thylefors B Chylack LT Jr Konyama K Sasaki K Sperduto R Taylor HR West S WHO Cataract Grading Group. A simplified cataract grading system. Ophthalmic Epidemiol 2002 9 83 95 11821974 10.1076/opep.9.2.83.1523

[15] Yoshitake Y Nishikawa K Production of monoclonal antibodies with specificity for different epitopes on the human epidermal growth factor molecule. Arch Biochem Biophys 1988 263 437 46 2454080 10.1016/0003-9861(88)90656-x

[16] Michael R Vrensen GF van Marle J Gan L Soderberg PG Apoptosis in the rat lens after in vivo threshold dose ultraviolet irradiation. Invest Ophthalmol Vis Sci 1998 39 2681 7 9856778

[17] Andley UP Song Z Mitchell DL DNA repair and survival in human lens epithelial cells with extended lifespan. Curr Eye Res 1999 18 224 30 10342377 10.1076/ceyr.18.3.224.5371

[18] Ibaraki N Ohara K Shimizu H Explant culture of human lens epithelial cells from senile cataract patients. Jpn J Ophthalmol 1993 37 310 7 8295370

[19] Yaar M Gilchrest BA Photoageing: mechanism, prevention and therapy. Br J Dermatol 2007 157 874 87 17711532 10.1111/j.1365-2133.2007.08108.x

[20] Rittiá L Fisher GJ UV-light-induced signal cascades and skin aging. Ageing Res Rev 2002 1 705 20 12208239 10.1016/s1568-1637(02)00024-7

[21] Shui YB Sasaki H Pan JH Hata I Kojima M Yamada Y Hirai KI Takahashia N Sasaki K Morphological observation on cell death and phagocytosis induced by ultraviolet irradiation in a cultured human lens epithelial cell line. Exp Eye Res 2000 71 609 18 11095913 10.1006/exer.2000.0917

[22] Shoyab M Plowman GD McDonald VL Bradley JG Todaro GJ Structure and function of human amphiregulin: a member of the epidermal growth factor family. Science 1989 243 1074 6 2466334 10.1126/science.2466334

[23] Singh B Schneider M Knyazev P Ullrich A UV-induced EGFR signal transactivation is dependent on proligand shedding by activated metalloproteases in skin cancer cell lines. Int J Cancer 2009 124 531 9 19003995 10.1002/ijc.23974

[24] Sternlicht MD Sunnarborg SW Kouros-Mehr H Yu Y Lee DC Werb Z Mammary ductal morphogenesis requires paracrine activation of stromal EGFR via ADAM17-dependent shedding of epithelial amphiregulin. Development 2005 132 3923 33 16079154 10.1242/dev.01966 PMC2771180

[25] Thompson SA Harris A Hoang D Ferrer M Johnson GR COOH-terminal extended recombinant amphiregulin with bioactivity comparable with naturally derived growth factor. J Biol Chem 1996 271 17927 31 8663535 10.1074/jbc.271.30.17927

[26] Pastore S Mascia F Mariani V Girolomoni G The epidermal growth factor receptor system in skin repair and inflammation. J Invest Dermatol 2008 128 1365 74 18049451 10.1038/sj.jid.5701184

[27] Willmarth NE Baillo A Dziubinski M Wilson K Rieseii D Ethier S Altered EGFR localization and degradation in human breast cancer cells with an amphiregulin/EGFR autocrine loop. Cell Signal 2009 21 212 9 18951974 10.1016/j.cellsig.2008.10.003 PMC2632975

[28] Berasain C Garcia-Trevijano ER Castillo J Erroba E Santamaria M Lee DC Prieto J Avila MA Novel role for amphiregulin in protection from liver injury. J Biol Chem 2005 280 19012 20 15753092 10.1074/jbc.M413344200

[29] Perugorria MJ Latasa MU Nicou A Cartagena-Lirola H Castillo J Goni S Vespasiani-Gentilucci U Zagami MG Lotersztajn S Prieto J Berasain C Avila MA The epidermal growth factor receptor ligand amphiregulin participates in the development of mouse liver fibrosis. Hepatology 2008 48 1251 61 18634036 10.1002/hep.22437

[30] Stoll S Garner W Elder J Heparin-binding ligands mediate autocrine epidermal growth factor receptor activation In skin organ culture. J Clin Invest 1997 100 1271 81 9276746 10.1172/JCI119641 PMC508305

[31] Rittié L Varani J Kang S Voorhees JJ Fisher GJ Retinoid-induced epidermal hyperplasia is mediated by epidermal growth factor receptor activation via specific induction of its ligands heparin-binding EGF and amphiregulin in human skin in vivo. J Invest Dermatol 2006 126 732 9 16470170 10.1038/sj.jid.5700202

[32] Bootcov MR Bauskin AR Valenzuela SM Moore AG Bansal M He XY Zhang HP Donnellan M Mahler S Pryor K Walsh BJ Nicholson RC Fairlie WD Por SB Robbins JM Breit SN MIC-1, a novel macrophage inhibitory cytokine, is a divergent member of the TGF-beta superfamily. Proc Natl Acad Sci USA 1997 94 11514 9 9326641 10.1073/pnas.94.21.11514 PMC23523

[33] Lawton LN Bonaldo MF Jelenc PC Qiu L Baumes SA Marcelino RA de Jesus GM Wellington S Knowles JA Warburton D Brown S Soares MB Identification of a novel member of the TGF-beta superfamily highly expressed in human placenta. Gene 1997 203 17 26 9426002 10.1016/s0378-1119(97)00485-x

[34] Paralkar VM Vail AL Grasser WA Brown TA Xu H Vukicevic S Ke HZ Qi H Owen TA Thompson DD Cloning and characterization of a novel member of the transforming growth factor-beta/bone morphogenetic protein family. J Biol Chem 1998 273 13760 7 9593718 10.1074/jbc.273.22.13760

[35] Johnen H Lin S Kuffner T Brown DA Tsai VW Bauskin AR Wu L Pankhurst G Jiang L Junankar S Hunter M Fairlie WD Lee NJ Enriquez RF Baldock PA Corey E Apple FS Murakami MM Lin EJ Wang C During MJ Sainsbury A Herzog H Breit SN Tumor-induced anorexia and weight loss are mediated by the TGF-beta superfamily cytokine MIC-1. Nat Med 2007 13 1333 40 17982462 10.1038/nm1677

[36] Ding Q Mracek T Gonzalez-Muniesa P Kos K Wilding J Trayhurn P Bing C Identification of macrophage inhibitory cytokine-1 in adipose tissue and its secretion as an adipokine by human adipocytes. Endocrinology 2009 150 1688 96 19074584 10.1210/en.2008-0952

[37] Dandrea T Hellmold H Jonsson C Zhivotovsky B Hofer T Warngard L Cotgreave I The transcriptosomal response of human A549 lung cells to a hydrogen peroxide-generating system: relationship to DNA damage, cell cycle arrest, and caspase activation. Free Radic Biol Med 2004 36 881 96 15019973 10.1016/j.freeradbiomed.2003.12.014

[38] Schlittenhardt D Schober A Strelau J Bonaterra GA Schmiedt W Unsicker K Metz J Kinscherf R Involvement of growth differentiation factor-15/macrophage inhibitory cytokine-1 (GDF-15/MIC-1) in oxLDL-induced apoptosis of human macrophages in vitro and in arteriosclerotic lesions. Cell Tissue Res 2004 318 325 33 15459768 10.1007/s00441-004-0986-3

[39] Akiyama M Okano K Fukada Y Okano T Macrophage inhibitory cytokine MIC-1 is upregulated by short-wavelength light in cultured normal human dermal fibroblasts. FEBS Lett 2009 583 933 7 19302795 10.1016/j.febslet.2009.02.006

[40] Tan M Wang Y Guan K Sun Y PTGF-beta, a type beta transforming growth factor (TGF-beta) superfamily member, is a p53 target gene that inhibits tumor cell growth via TGF-beta signaling pathway. Proc Natl Acad Sci USA 2000 97 109 14 10618379 10.1073/pnas.97.1.109 PMC26624

[41] Hromas R Hufford M Sutton J Xu D Li Y Lu L PLAB, a novel placental bone morphogenetic protein. Biochim Biophys Acta 1997 1354 40 4 9375789 10.1016/s0167-4781(97)00122-x

[42] Xu J Kimball TR Lorenz JN Brown DA Bauskin AR Klevitsky R Hewett TE Breit SN Molkentin JD GDF15/MIC-1 functions as a protective and antihypertrophic factor released from the myocardium in association with SMAD protein activation. Circ Res 2006 98 342 50 16397142 10.1161/01.RES.0000202804.84885.d0

[43] Kempf T Eden M Strelau J Naguib M Willenbockel C Tongers J Heineke J Kotlarz D Xu J Molkentin JD Niessen HW Drexler H Wollert KC The transforming growth factor-beta superfamily member growth-differentiation factor-15 protects the heart from ischemia/reperfusion injury. Circ Res 2006 98 351 60 16397141 10.1161/01.RES.0000202805.73038.48

